# Effect of exercise training on the FNDC5/BDNF pathway in spontaneously hypertensive rats

**DOI:** 10.14814/phy2.14323

**Published:** 2019-12-27

**Authors:** Tao Wang, Melissa T. Maltez, Heow Won Lee, Monir Ahmad, Hong‐Wei Wang, Frans H. H. Leenen

**Affiliations:** ^1^ Brain and Heart Research Group University of Ottawa Heart Institute Ottawa ON Canada; ^2^ Present address: Toronto General Hospital Research Institute University Health Network Toronto ON Canada

**Keywords:** BDNF, brain, exercise, heart, hypertension, skeletal muscle

## Abstract

Increased sympathetic activity contributes to the development of cardiovascular diseases such as hypertension. Exercise training lowers sympathetic activity and is beneficial for the prevention and treatment of hypertension and associated cognitive impairment. Increased BDNF expression in skeletal muscle, heart, and brain may contribute to these actions of exercise, but the mechanisms by which this occurs are unknown. We postulated that hypertension is associated with decreased hippocampal BDNF, which can be restored by exercise‐mediated upregulation of fibronectin type‐II domain‐containing 5 (FNDC5). Spontaneously hypertensive rats (SHR) and normotensive Wistar–Kyoto rats (WKY) were subjected to 5 weeks of motorized treadmill training. BDNF and FNDC5 expressions were measured in the left ventricle (LV), quadriceps, soleus muscle, and brain areas. Exercise training reduced blood pressure (BP) in both strains. BDNF and FNDC5 protein in the LV were increased in SHR, but exercise increased only BDNF protein in both strains. BDNF mRNA, but not protein, was increased in the quadriceps of SHR, and BDNF mRNA and protein were decreased by exercise in both groups. FNDC5 protein was higher in SHR in both the quadriceps and soleus muscle, whereas exercise increased FNDC5 protein only in the quadriceps in both strains. BDNF mRNA was lower in the dentate gyrus (DG) of SHR, which was normalized by exercise. BDNF mRNA expression in the DG negatively correlated with BP. No differences in FNDC5 expression were observed in the brain, suggesting that enhanced BDNF signaling may contribute to the cardiovascular and neurological benefits of exercise training, and these processes involve peripheral, but not central, FNDC5.

## Graphical abstract

BDNF in the LV and quadriceps were higher in SHR, with increases in FNDC5. Exercise increased BDNF in the LV and decreased BDNF in the quadriceps. Hippocampal BDNF decreased in SHR, but normalized with exercise. FNDC5 was increased by exercise only in skeletal muscle, suggesting a role for peripheral, not central, FNDC5 in inducing BDNF, which may contribute to the benefits of exercise.

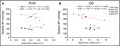

## 1 INTRODUCTION

Regular moderate‐ to high‐intensity exercise is beneficial for the prevention and treatment of cardiovascular disease (CVD) and CVD risk factors, such as hypertension (Lavie et al., 2015; Lear et al., 2017). The anti‐hypertensive benefits of exercise are governed by both peripheral and central mechanisms of blood pressure (BP) control. However, the mechanisms by which exercise induces these changes remain unclear.

Exercise‐induced decrease in sympathetic activity may be mediated—at least in part—by brain‐derived neurotrophic factor (BDNF) (Garcia, Chen, Garza, Cotman, & Russo‐Neustadt, 2003; Walsh & Tschakovsky, 2018). BDNF is secreted by both the brain and skeletal muscle in response to physical activity (Liu & Nusslock, 2018; Matthews et al., 2009). BDNF and its receptor tropomyosin‐related receptor kinase B (TrkB) are critically involved in cognitive function, synaptic plasticity, and neuronal survival (Greenberg, Xu, Lu, & Hempstead, 2009; Liu & Nusslock, 2018; Park & Poo, 2013). In the brain, exercise‐induced increase in hippocampal BDNF promotes neurogenesis and improves cognition (Liu & Nusslock, 2018), while conversely, BDNF deficiency contributes to cognitive impairment and neurodegenerative diseases (Zuccato & Cattaneo, 2009). In the heart, there is growing evidence for an important role for BDNF‐TrkB signaling in the proper development of the heart and its vasculature (Kermani & Hempstead, 2019; Pius‐Sadowska & Machalinski, 2017), cardiac inotropy and lusitropy (Feng et al., 2015), and improving cardiac function postmyocardial infarction (MI) (Lee, Ahmad, Wang, & Leenen, 2017; Lee et al., 2018). In skeletal muscle, it contributes to muscle regeneration (Yu, Chang, Gao, Li, & Zhao, 2017) and fat metabolism (Matthews et al., 2009). BDNF also modulates cardiovascular function through effects in cardiovascular regulatory nuclei in the brain, such as the paraventricular nucleus (PVN) and the rostral ventrolateral medulla (RVLM) (Becker, Wang, Tian, & Zucker, 2015; Chan, Wu, Chang, Hsu, & Chan, 2010; Schaich, Wellman, Einwag, Dutko, & Erdos, 2018). BDNF may do so through angiotensinergic signaling pathways involved in the regulation of BP (Becker et al., 2015; Chan et al., 2010; Erdos, Backes, McCowan, Hayward, & Scheuer, 2015; Schaich et al., 2018). Spontaneously hypertensive rats (SHR), a rodent model of genetic essential hypertension in humans, have markedly reduced BDNF expression in the RVLM compared to normotensive Wistar–Kyoto (WKY) rats, which is dependent on reactive oxygen species (Chan et al., 2010). Infusion of BDNF into the cisterna magna significantly lowered BP of SHR (Chan et al., 2010). SHR also exhibit elevated levels of proinflammatory cytokines and increased expression of AT_1_R and angiotensin‐converting enzyme in the PVN and RVLM (Agarwal, Welsch, Keller, & Francis, 2011), as well as higher expression of the mineralocorticoid receptors (MR) (Pietranera et al., 2012). A moderate intensity exercise program reduced the elevated proinflammatory cytokines and AT_1_R expression in the PVN and RVLM and also reduced BP (Agarwal et al., 2011). While BDNF expression has been implicated in BP control, it is unclear how exercise might elicit changes in these systems.

One potential mechanism by which exercise may increase BDNF is through myokines such as irisin, which are secreted by skeletal muscle into the circulation following exercise and then signal to the brain (Delezie & Handschin, 2018; Fiuza‐Luces et al., 2018). Irisin—the cleaved protein product of fibronectin type‐II domain‐containing 5 (FNDC5) expressed abundantly in heart, skeletal muscle, and brain—has been associated with beneficial effects on the hypertensive vasculature (Fu et al., 2016; Ling et al., 2018), cardiac remodeling and fibrosis (Chen et al., 2019; Liao et al., 2019; Yu et al., 2019), and metabolism (Bostrom et al., 2012; Lee et al., 2014). FNDC5 is expressed downstream of the master skeletal muscle regulator peroxisome proliferator‐activated receptor coactivator 1‐alpha (PGC1α), and following exercise training, the activation of the PGC1α/FNDC5 pathway leads to increased BDNF expression in the hippocampus (Wrann et al., 2013). Whether FNDC5 might be involved in the beneficial effects of exercise in hypertension remains to be determined.

Thus, we postulated that hypertension is associated with downregulation of BDNF‐TrkB signaling in the hippocampus, which can be rescued by exercise training through the upregulation of FNDC5 expression in skeletal muscle and brain. In this study, we subjected SHR and normotensive control WKY to a moderate‐intensity exercise treadmill program for 5 weeks. The heart (LV), skeletal muscle (quadriceps and soleus muscles), and brain were collected for the measurement of BDNF, TrkB, and FNDC5 mRNA and protein expression. Our findings demonstrate that endurance exercise training increases BDNF‐TrkB‐signaling in the heart and the dentate gyrus (DG) of the hippocampus and increases FNDC5 expression in the quadriceps but not the heart and brain.

## 2 METHODS

### 2.1 Ethical approval

All experiments were approved by the University of Ottawa Animal Care Committee, and conform to the *Guide for the Care and Use of Laboratory Animals* published by the US National Institutes of Health (8th edn, 2011).

### 2.2 Animals

Male SHR (*n* = 15) and WKY (*n* = 17), aged 5–6 weeks and weighing 130–170 g, were obtained from Charles River Breeding Laboratories and acclimatized for 5–7 days in a room maintained at constant temperature and humidity. Rats were housed in pairs under a 12:12‐hr light–dark cycle and allowed standard laboratory chow and tap water ad libitum*.*

### 2.3 Exercise training

Rats were randomly divided into four groups: sedentary WKY (WKY‐Sed), exercise‐trained WKY (WKY‐ExT), sedentary SHR (SHR‐Sed), or exercise‐trained SHR (SHR‐ExT). A treadmill exercise program was conducted for 5 weeks, 5 days/week in the afternoon as previously described (Lee et al., 2017; Zheng, Sharma, Liu, & Patel, 2012). Rats were acclimatized to running on a motor‐driven treadmill (Columbus Instruments) at low speed (10 m/min), grade 0%, and short duration (10–15 min/day) for the first 3 days. For the remainder of week 1 and week 2, the speed, grade, and duration were gradually increased to 15–20 m/min, 5%–10% incline, and 60 min/day, respectively, which is considered moderate intensity for rats (Zheng et al., 2012). These exercise parameters were maintained at the maximal level for weeks 3, 4, and 5. In every session from week 2 onward, a 5‐min warm‐up was conducted at low speed (10 m/min) and 0% inclines which was not included in the total duration of the exercise training session. Two WKY rats refused to run and were removed from the exercise group. Sedentary groups were handled daily under the same conditions as the exercise‐trained groups and exposed to the immobile treadmill for comparable times. Body weight was measured at the beginning of each week, as well as after the final exercise session of week 5 prior to hemodynamic measurements.

### 2.4 Blood pressure measurements

Rats were placed into individual cages 24 hr prior to cannulation. In the morning, under anesthesia with 2.5% isoflurane in oxygen, the right femoral artery was cannulated using PE50‐10 tubing, filled with heparinized saline. Cannulae were secured by a stopper pin and exteriorized to the back. Rats were then individually housed in a quiet room near the data acquisition system. In the afternoon, intra‐arterial cannulae were connected to pressure transducers. After a rest period of 30 min, BP and heart rate (HR) were recorded for a period of 10 min using AcqKnowledge 3.8 software (Biopac Systems Inc.).

### 2.5 Tissue collection

After sacrifice by decapitation, the brain was removed and immediately frozen in chilled methylbutane (Sigma‐Aldrich) and then stored at –80°C. The heart was immediately rinsed in ice‐cold 0.9% saline. Working rapidly on ice, the right ventricle (RV) was separated from the left ventricle (LV) then blotted dry, weighed, and frozen in liquid nitrogen. Soleus muscle and quadriceps from the noncannulated left hind leg were collected, blotted dry, weighed, and frozen in liquid nitrogen. Trunk blood was collected into prechilled heparinized falcon tubes and plasma was isolated by centrifugation. Top‐most layer of plasma was centrifuged again and collected for BDNF analysis. The remaining plasma was aliquoted for measurement of pro‐ and anti‐inflammatory cytokines. Plasma samples were immediately stored at −80°C.

### 2.6 Real‐time qPCR

Samples were homogenized in 1 ml (per 100 mg tissue) QIAzol Lysis Reagent (Qiagen Inc.) using a prechilled Polytron, and RNA was isolated as per manufacturer's instructions. To eliminate potential genomic DNA contamination, a quantity of 20 µg total RNA was treated with DNase I (Invitrogen). A quantity of 5 µg of DNase I‐treated RNA was used for cDNA synthesis by incubation with 200 U/µl RevertAid H Minus reverse transcriptase and Oligo‐dT as primer (Thermo Scientific Inc.) at 42°C for 60 min.

For brain RNA, serial 50‐µm coronal‐oriented brain cryosections were obtained, and brain nuclei were collected as described (Wang et al., 2016). Briefly, brain tissue was collected from −0.80 to −3.14 mm and −11.80 to −13.80 mm posterior to bregma for the forebrain and hindbrain, respectively, based on the rat brain atlas of Paxinos & Watson (1998). The PVN, CA1‐CA3, and DG regions of the hippocampus, and RVLM were extracted via micro‐punch and homogenized immediately in ice‐cold lysis buffer (ReliaPrep RNA tissue mini‐prep system, Promega). Total RNA was isolated according to the manufacturer's instruction*.* A quantity of 300–500 ng of DNase I‐treated RNA was used for cDNA synthesis by Verso cDNA kit (Thermo Scientific Inc.).

Primers of BNDF (NM‐012513.4), TrkB (NM‐001163168.2), FNDC5 (NM‐001270981.1), AT_1_R (NM‐030985.4), and MR (NM‐013131.1) were designed based on published Genbank sequences, respectively. The sequences of the primers are as follows: BDNF (*Forward: 5′‐ CGAGACCAAGTGTAATCCCATG‐3; Reverse: 5′‐CAGGAAGTGTCTATCCTTATGAACC‐3′*), TrkB (*Forward: 5′‐CAAGACTCTGTGAACCTCACTG‐3′; Reverse: 5′‐TCCGTGTGATTGGTGACGTGTA‐3′*), FNDC5 (*Forward: 5′‐CAGCAGAAGAAGGATGTGAG‐3′; Reverse: 5′‐GGCAGAAGAGAGCTATGACA‐3′*), AT_1_R (Forward: 5*′*‐*GCACACTGGCAATGTAATGC*‐3*′*, Reverse: 5*′*‐*GTTGAACAGAACAAGTGACC*‐3*′*), and MR (Forward: 5*′*‐*GCTCAACATTGTCCAGTACA*‐3*′*, Reverse: 5*′*‐*GCACAGGTGGTCCTAAGAGATT*‐3*′*).

For FNDC5, a 316‐bp PCR fragment corresponding to position 208–523, BDNF, a 165‐bp fragment at 1212–1372 position, and TrkB, a 197‐bp fragment at 1502–1698, were amplified and then subcloned into pCRII‐TA vector (Invitrogen) followed by restriction endonuclease analysis. The concentrations of the plasmids were determined via NanoDrop spectrophotometry at 260 nm. An external standard curve for each plasmid was created using serial 10‐fold dilutions (eg. 100 pg/µl to 0.001 pg/µl) of plasmid under the same real‐time qPCR conditions described above.

Real‐time qPCR was performed with a Roche LightCycler LC480 using LC480 SYBR Green I (Roche Diagnostics). PCR conditions were set as follows: initial at 95°C for 10 s followed by 45 cycles of denaturation at 95°C for 10 s, annealing for 15 s at 57°C for FNDC5 and BDNF, 59°C for TrKB, and 62°C for AT_1_R, and MR, followed by extension at 72°C. Specificity of the real‐time qPCR products was determined by both melting curve analysis and agarose gel electrophoresis. mRNA abundance of various genes was normalized against phosphoglycerate kinase 1 (PGK1) levels as the endogenous reference. The external standard of PGK1 was the same as previously described (Wang et al., 2010).

### 2.7 Western blot

Tissue samples were homogenized in 1 ml (per 100 mg tissue) modified RIPA buffer (50 mM Tris pH 8.0, 150 mM NaCl, 0.5% sodium deoxycholate, 1% NP‐40, 1 mM EDTA pH 8.0, and 0.1% SDS, with protease inhibitor cocktail (Sigma‐Aldrich) and phosphatase inhibitors 10 mM sodium fluoride and 2 mM sodium orthovanadate). Protein was isolated from homogenates by centrifugation at 10,000*g* for 30 min at 4°C, and the protein concentration was measured by BCA assay (Thermo Scientific Inc.).

A quantity of 20–50 μg of total protein was loaded for western blotting. Proteins were separated in SDS‐PAGE gels and transferred onto PVDF membranes (Bio‐Rad), then blocked with 5% milk in TBS with 0.1% Tween‐20. The following primary and secondary antibodies were used: anti‐BDNF (1:3,000, Abcam: cat#ab108319), anti‐TrkB (1:1,000, Millipore: cat#07‐225), anti‐FNDC5 (1:3,000, Abcam: cat#ab174833), anti‐GAPDH (1:10,000, Millipore: cat#MAB374), anti‐rabbit (1:5,000, Jackson ImmunoResearch: cat#111‐035‐144), anti‐mouse (1:10,000, GE Healthcare UK Limited: cat#NXA931V). The membranes were developed with either Immobilon Forte Western HRP substrate (BDNF and FNDC5) or Immobilon Classico Western HRP substrate (TrKB and GAPDH) (Millipore) and visualized with an Alpha Innotech Imager (Fluorchem 9900. Alpha Innotech). Following densitometric analysis using AlphaEase (Alpha Innotech) software, relative protein expression was calculated as the target protein band density normalized to the endogenous reference protein GAPDH band density in the same sample.

### 2.8 Biochemical plasma assays

Total BDNF (pro‐BDNF plus mature BDNF) in the plasma was measured with a commercially available ELISA kit (ChemiKine, Cat#CYT306, EMD Millipore) according to manufacturer's instructions. Plasma samples were diluted 1:1 with a sample buffer provided by the kit and run in duplicate. Total BDNF levels are expressed as pg/ml of plasma.

To measure plasma pro‐ and anti‐inflammatory cytokine levels, a commercially available rat cytokine 12‐plex Bio‐Plex assay kit (Bio‐Rad) was used according to manufacturer's instructions. Plasma samples were diluted 1:4 with a sample buffer provided by the kit and run in singlicate. Cytokine levels are expressed as pg/ml of plasma.

### 2.9 Statistical analysis

All values are expressed as mean ± *SD*. Two‐way ANOVA was performed to determine the effects of both exercise and hypertension on the various parameters using IBM SPSS statistics 25 software. When significant differences were found between groups, a student's Newmans–Keuls test was run for post hoc comparative analysis. Correlations analyses were done by Pearson's correlation analysis. Statistical significance was defined as *p* < .05.

## 3 RESULTS

### 3.1 Effect of exercise on BP, HR, and cardiac weights

As expected, SHR had significantly higher SBP, DBP, and HR when compared to WKY rats (Table 1). Following exercise, SBP was 7 mmHg lower in WKY‐ExT versus WKY‐Sed and 28 mmHg lower in SHR‐ExT versus SHR‐Sed. DBP was 10 mmHg lower in WKY‐ExT versus WKY‐Sed and 7 mmHg lower in SHR‐ExT versus SHR‐Sed. HR was unaffected by exercise (Table 1).

**Table 1:** Systolic (SBP) and diastolic blood pressure (DBP), heart rate (HR), left ventricle (LV) and right ventricle (RV) weights, and final body weight of SHR and WKY with or without exercise training

	WKY		SHR	
	Sed (*n* = 10)	ExT (*n* = 7)	Sed (*n* = 6)	ExT (*n* = 9)
SBP (mmHg)	147 ± 7	140 ± 4†	202 ± 13*	184 ± 7*†
DBP (mmHg)	100 ± 7	90 ± 5†	134 ± 5*	127 ± 5*†
HR (bpm)	378 ± 43	376 ± 29	452 ± 34*	442 ± 25*
LV (mg/100 g BW)	245 ± 14	252 ± 8	287 ± 6*	284 ± 17*
RV (mg/100 g BW)	61 ± 23	58 ± 2	54 ± 6	51 ± 8
Final BW (g)	248 ± 9	263 ± 7	270 ± 9*§	264 ± 18*
Total weight gain (g)	111 ± 8	99 ± 25	99 ± 18	104 ± 20

Note

Values are means ± *SD*. For SBP, SHR versus WKY, *F* = 258.31, *p* < .001; ExT versus Sed, *F* = 16.72, *p* < .001; ExT × strain interaction, *F* = 2.96, *p* = .10 NS. For DBP, SHR versus WKY, *F* = 270.99, *p* < .001; ExT versus Sed, *F* = 15.07, *p* < .001; ExT × strain interaction, *F* = .58, *p* = .46 NS. For HR, SHR versus WKY, *F* = 28.58, *p* < .001; ExT versus Sed, *F* = .20, *p* = .66 NS; ExT × strain interaction, *F* = .07, *p* = .79 NS. For LV weight, SHR versus WKY, *F* = 46.14, *p* < .001; ExT versus Sed, *F* = .16, *p* = .70 NS; ExT × strain interaction, *F* = .98, *p* = .33 NS. For RV weight, SHR versus WKY, *F* = 1.35, *p* = .26 NS; ExT versus Sed, *F* = .21, *p* = .65 NS; ExT × strain interaction, *F* = .000011, *p* = 1.00 NS. For Final BW, SHR versus WKY, *F* = 6.26, *p* = .019; ExT versus Sed, *F* = .81, *p* = .38 NS; ExT × strain interaction, *F* = 5.32, *p* = .029. For total weight gain, SHR versus WKY, *F* = .25, *p* = .62 NS; ExT versus Sed, *F* = .24, *p* = .63 NS; ExT × strain interaction, *F* = 1.60, *p* = .22 NS.

* *p* < .05 versus WKY.

^†^ *p* < .05 versus Sed.

^§^ *p* < .05 versus WKY‐Sed.

SHR had significantly higher body weight compared to WKY, which was notable in SHR‐Sed versus WKY‐Sed (Table 1). LV weight was larger in SHR when compared to WKY, while RV weight was similar. Exercise had no significant effect on LV and RV weights (Table 1). Thus, we demonstrate that SBP and DBP in both hypertensive and normotensive rats were significantly decreased following 5 weeks of endurance exercise training.

### 3.2 Effect of hypertension and exercise on plasma BDNF and cytokines

Plasma BDNF tended (*p* = .09) to be lower in SHR than WKY and was not affected by exercise in either strain (Table 2). The anti‐inflammatory cytokine IL‐13 was significantly elevated in SHR versus WKY and tended (*p* = .09) to be higher following exercise in SHR‐ExT versus SHR‐Sed. The anti‐inflammatory cytokine IL‐4 was similar in SHR versus WKY, but tended (*p* = .08) to be higher following exercise. The proinflammatory cytokine IL‐1α was similar between SHR versus WKY, but tended (*p* = .07) to be lower following exercise in WKY‐ExT versus WKY‐Sed. No other cytokines were affected by strain or exercise (Table 2).

**Table 2:** Plasma BDNF and cytokines in SHR and WKY with or without exercise training

	WKY		SHR	
	Sed (*n* = 10)	ExT (*n* = 7)	Sed (*n* = 6)	ExT (*n* = 9)
BDNF (pg/mL)	50 ± 10	51 ± 5	46 ± 5	46 ± 5
Anti‐inflammatory cytokines				
IL‐4 (pg/ml)	1.3 ± 0.4	0.9 ± 0.5	0.9 ± 0.8	2.3 ± 2
IL‐5 (pg/ml)	24 ± 9	25 ± 6	21 ± 6	28 ± 12
IL‐10 (pg/ml)	33 ± 9	33 ± 6	29 ± 6	36 ± 7
IL‐13 (pg/ml)	0.7 ± 0.6	0.3 ± 0.3	0.8 ± 0.0*	1.4 ± 0.8*
Pro‐inflammatory cytokines				
IL‐1α (pg/ml)	9.1 ± 5	5.4 ± 4	5.4 ± 3	6.9 ± 3
IL‐1β (pg/ml)	28 ± 5	25 ± 3	24 ± 8	26 ± 6
TNF‐α (pg/ml)	8.0 ± 3	7.3 ± 2	7.7 ± 3	7.6 ± 3

Note

Values are means ± *SD*. For plasma BDNF, SHR versus WKY, *F* = 3.13, *p* = .09 NS; ExT versus Sed, *F* = .07, *p* = .80 NS; ExT × strain interaction, *F* = .002, *p* = .97 NS. For IL‐4, SHR versus WKY, *F* = .80, *p* = .38 NS; ExT versus Sed, *F* = 3.36, *p* = .08 NS; ExT × strain interaction, *F* = 1.86, *p* = .19 NS. For IL‐5, SHR versus WKY, *F* = .003, *p* = .95 NS; ExT versus Sed, *F* = 1.55, *p* = .22 NS; ExT × strain interaction, *F* = 1.11, *p* = .30 NS. For IL‐10, SHR versus WKY, *F* = .006, *p* = .94 NS; ExT versus Sed, *F* = 1.05, *p* = .32 NS; ExT × strain interaction, *F* = .98, *p* = .33 NS. For IL‐13, SHR versus WKY, *F* = 4.53, *p* = .047; ExT versus Sed, *F* = .11, *p* = .75 NS; ExT × strain interaction, *F* = 3.09, *p* = .10 NS. For IL‐1α, SHR versus WKY, *F* = .68, *p* = .42 NS; ExT versus Sed, *F* = .73, *p* = .40 NS. ExT × strain interaction, *F* = 3.63, *p* = .07 NS. For IL‐1β, SHR versus WKY, *F* = .32, *p* = .58 NS; ExT versus Sed, *F* = .02, *p* = .88 NS. ExT × strain interaction, *F* = 1.69, *p* = .21 NS. For TNF‐α, SHR versus WKY, *F* = .0004, *p* = .98 NS; ExT versus Sed, *F* = .22, *p* = .65 NS. ExT × strain interaction, *F* = .11, *p* = .75 NS.

* *p* < .05 versus WKY.

### 3.3 Effects of hypertension and exercise on BDNF, TrkB, and FNDC5 mRNA and protein expression in the heart and skeletal muscle

**Figure 1:**
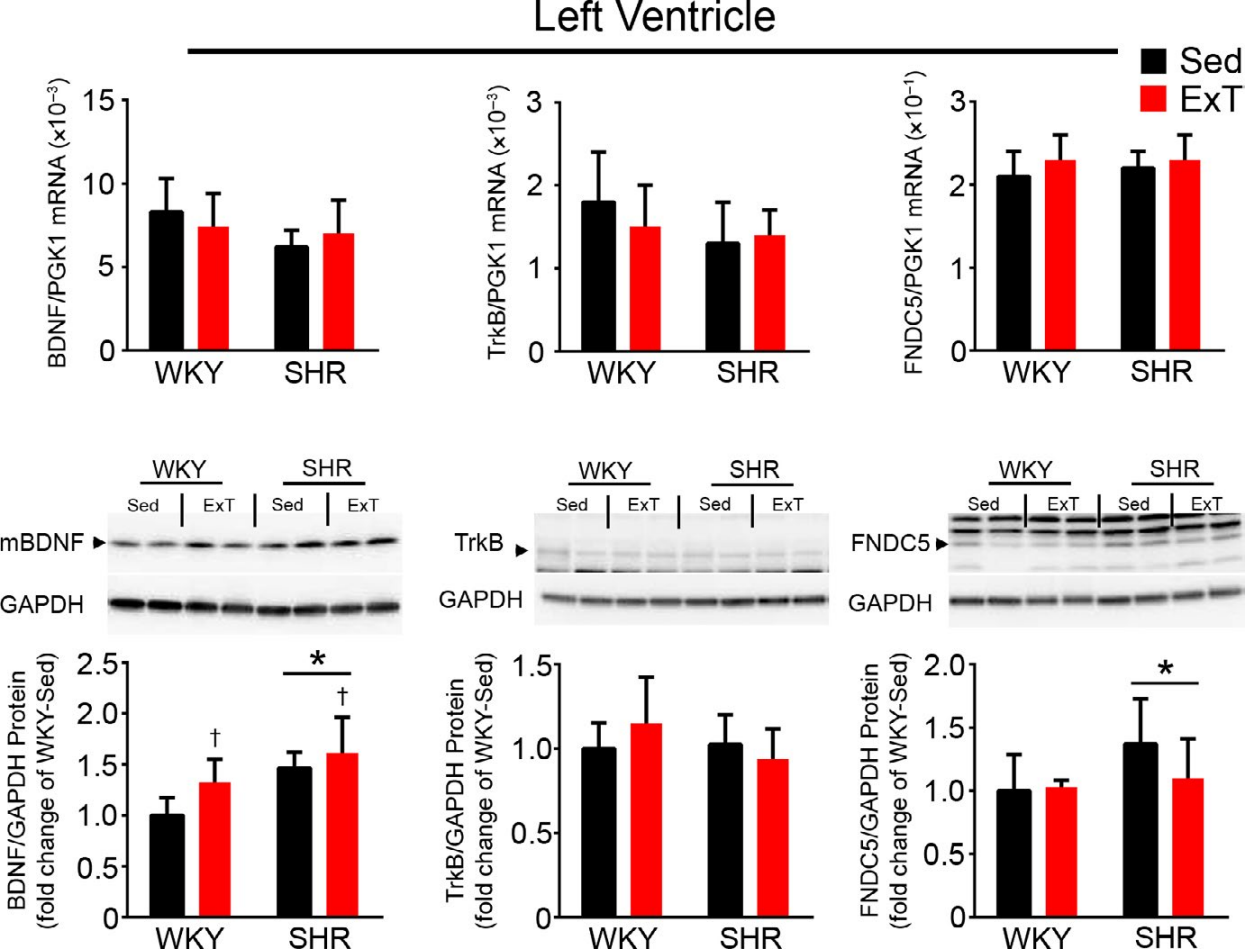
BDNF, TrkB, and FNDC5 mRNA, and protein expression in the LV of SHR and WKY with or without 5 weeks of exercise training. Upper panel shows summary data of mRNA in the LV. Lower panel represents representative western blot images and summary data of protein in bar graphs. Values of different proteins in the WKY‐Sed were normalized to 1. Values are mean ± *SD* (*n* = 7–10/group). For LV BDNF mRNA: SHR versus WKY, *F* = 3.85, *p* = .06 NS; ExT versus Sed, *F* = .003, *p* = .95 NS; ExT × strain interaction, *F* = 1.69, *p* = .21 NS. For LV BDNF protein: SHR versus WKY, *F* = 16.85, *p* = .0004; ExT versus Sed, *F* = 6.68, *p* = .016; ExT × strain interaction, *F* = .92, *p* = .35 NS. For LV TrkB mRNA: SHR versus WKY, *F* = 3.13, *p* = .09 NS; ExT versus Sed, *F* = .31, *p* = .85 NS; ExT × strain interaction, *F* = 1.76, *p* = .20 NS. For LV TrkB protein: SHR versus WKY, *F* = 1.62, *p* = .22 NS; ExT versus Sed, *F* = .18, *p* = .68 NS; ExT × strain interaction, *F* = 2.62, *p* = .12 NS. For LV FNDC5 mRNA: SHR versus WKY, *F* = .83, *p* = .37 NS; ExT versus Sed, *F* = 1.92, *p* = .18 NS; ExT × strain interaction, *F* = .26, *p* = .61 NS. For LV FNDC5 protein: SHR versus WKY, *F* = 4.69, *p* = .04; ExT versus Sed, *F* = 1.44, *p* = .24 NS; ExT × strain interaction, *F* = 2.25, *p* = .15 NS. *p < .05 versus WKY. ^†^ *p* < .05 versus Sed

In the LV, BDNF mRNA expression tended (*p* = .06) to be lower in SHR versus WKY, but protein expression was higher in SHR versus WKY and significantly elevated by exercise training in both SHR and WKY. TrkB mRNA expression tended (*p* = .09) to be lower in SHR versus WKY, while protein expression was similar. FNDC5 mRNA expression was similar, but protein expression was significantly higher in SHR compared to WKY (Figure 1). TrKB and FNDC5 in the LV were not changed by exercise.

**Figure 2:**
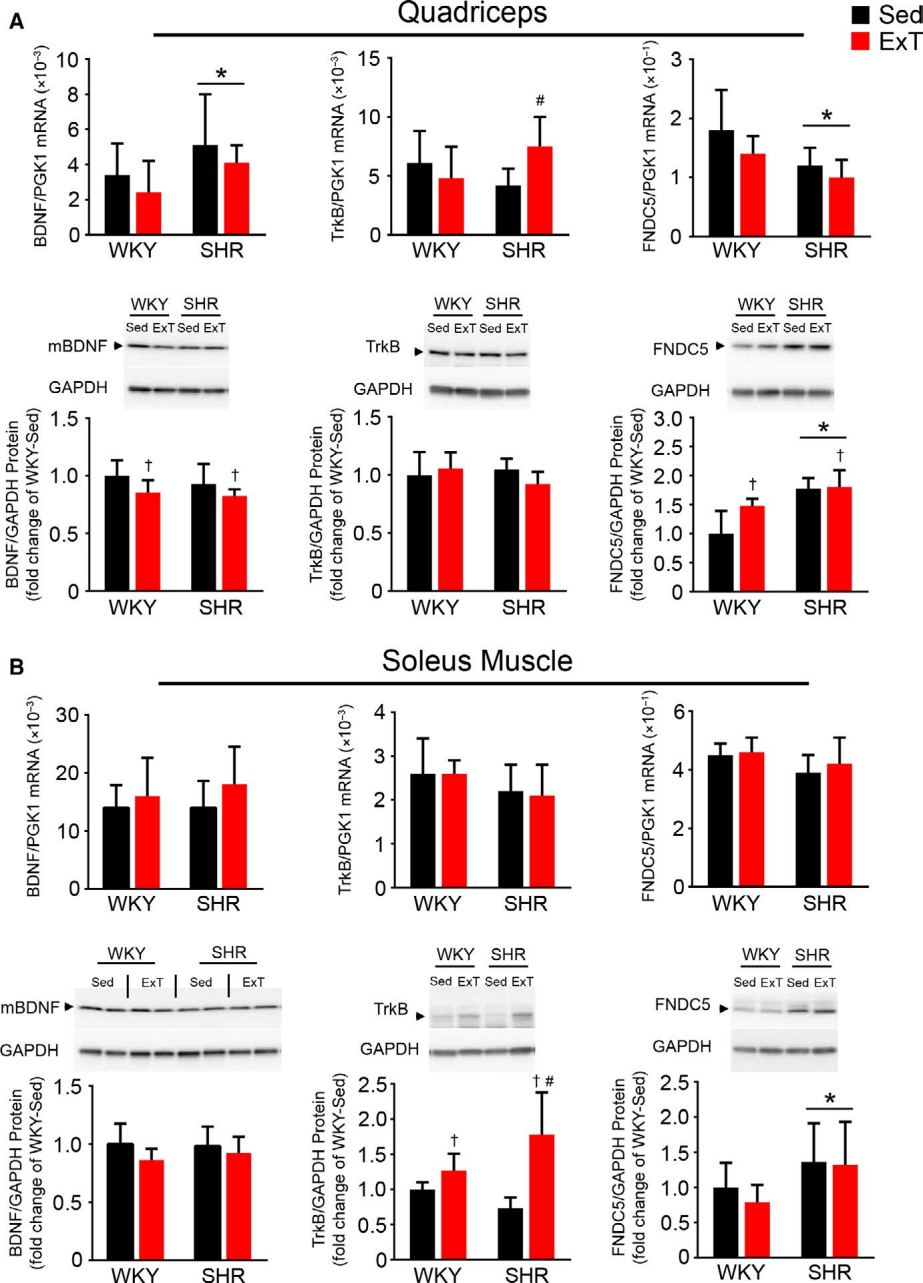
BDNF, TrkB, and FNDC5 mRNA and protein expression in the quadriceps and soleus muscle of SHR and WKY with or without 5 weeks of exercise training. BDNF, TrkB, and FNDC5 mRNA and protein in the (A) quadriceps and (B) soleus muscle. Representative western blot images are shown. Values are mean ± *SD* (*n* = 7–10/group). For quadriceps BDNF mRNA: SHR versus WKY, *F* = 5.21, *p* = .03; ExT versus Sed, *F* = 1.67, *p* = .21 NS; ExT × strain interaction, *F* = .005, *p* = .94 NS. For quadriceps BDNF protein: SHR versus WKY, *F* = .74, *p* = .40 NS; ExT versus Sed, *F* = 5.15, *p* = .04; ExT × strain interaction, *F* = .15, *p* = .71 NS. For quadriceps TrkB mRNA: SHR versus WKY, *F* = .31, *p* = .58 NS; ExT versus Sed, *F* = .73, *p* = .40 NS; ExT × strain interaction, *F* = 4.92. *p* = .03; For quadriceps TrkB protein: SHR versus WKY, *F* = .47, *p* = .50 NS; ExT versus Sed, *F* = .35, *p* = .56 NS; ExT × strain interaction, *F* = 2.39, *p* = .14 NS; For quadriceps FNDC5 mRNA: SHR versus WKY, *F* = 6.23, *p* = .02; ExT versus Sed, *F* = 2.81, *p* = .11 NS; ExT × strain interaction, *F* = .23, *p* = .64 NS. For quadriceps FNDC5 protein: SHR versus WKY, *F* = 5.15, *p* = .035; ExT versus Sed, *F* = 24.11, *p* < .0001; ExT × strain interaction, *F* = 3.99, *p* = .06 NS. For soleus muscle BDNF mRNA: SHR versus WKY, *F* = .40, *p* = .53 NS; ExT versus Sed, *F* = 2.95, *p* = .10 NS; ExT × strain interaction, *F* = .30, *p* = .59 NS. For soleus muscle BDNF protein: SHR versus WKY, *F* = .14, *p* = .71 NS; ExT versus Sed, *F* = 2.89, *p* = .10 NS; ExT × strain interaction, *F* = .46, *p* = .51 NS. For soleus muscle TrkB mRNA: SHR versus WKY, *F* = 3.38, *p* = .08 NS; ExT versus Sed, *F* = .031, *p* = .86 NS; ExT × strain interaction, *F* = .13, *p* = .72 NS. For soleus muscle TrkB protein: SHR versus WKY, *F* = .81, *p* = .38 NS. ExT versus Sed, *F* = 23.04, *p* < .001; ExT × strain interaction, *F* = 8.01, *p* = .010; For soleus muscle FNDC5 mRNA: SHR versus WKY, *F* = 2.95, *p* = .10 NS; ExT versus Sed, *F* = .51, *p* = .48 NS; ExT × strain interaction, *F* = .13, *p* = .73 NS. For soleus muscle FNDC5 protein: SHR versus WKY, *F* = 6.31, *p* = .02; ExT versus Sed, *F* = .50, *p* = .49 NS; ExT × strain interaction, *F* = .23, *p* = .64 NS. * *p* < .05 versus WKY. ^†^ *p* < .05 versus Sed. #*p* < .05 versus WKY‐ExT or SHR‐Sed

In the quadriceps, BDNF mRNA was significantly higher in SHR versus WKY, but BDNF protein was similar. BDNF protein expression was moderately reduced in both strains following exercise training. TrkB mRNA was similar in sedentary SHR and WKY and was elevated in SHR‐ExT versus SHR‐Sed group, while protein expression was similar among groups. FNDC5 mRNA was moderately lower while protein expression was significantly higher in SHR versus WKY. FNDC5 protein, but not mRNA, was elevated by exercise training in WKY (Figure 2a).

In the soleus muscle, BDNF expression was similar between strains, and exercise tended (*p* = .097) to increase BDNF mRNA but not protein. TrkB mRNA tended (*p* = .077) to be lower in SHR, whereas protein expression was elevated by exercise training in both strains. FNDC5 mRNA tended (*p* = .097) to be lower in SHR, but protein expression was higher in SHR (Figure 2b). Neither was affected by exercise.

### 3.4 Effects of exercise on BDNF, TrkB, FNDC5, AT_1_R, and MR mRNA expression in the brain of SHR versus WKY

We next examined the effects of exercise on BDNF, TrkB, and FNDC5 mRNA expression in the PVN and RVLM of SHR versus WKY. To evaluate the potential effects on angiotensinergic signaling, we also evaluated the expression of AT_1_R and MR (Table 3). In the PVN, TrkB expression was elevated in SHR versus WKY, but BDNF, FNDC5, AT_1_R, and MR expressions were similar in the two strains. Exercise had no effect on their expressions. In the RVLM, only MR was significantly lower in SHR versus WKY, and tended (*p* = .053) to be lowered by exercise in both strains. BDNF, TrkB, FNDC5, and AT_1_R expression were similar in the two strains with no effect of exercise.

**Table 3:** mRNA expression of BDNF, TrkB, FNDC5, AT_1_R, and MR in brain areas of SHR and WKY with or without exercise training

	WKY		SHR	
	Sed (*n* = 10)	ExT (*n* = 7)	Sed (*n* = 6)	ExT (*n* = 9)
BDNF				
PVN mRNA/PGK1 (×10^–2^)	4.3 ± 0.5	3.7 ± 0.3	4.0 ± 0.4	4.2 ± 0.3
RVLM mRNA/PGK1 (×10^–2^)	1.6 ± 0.2	1.7 ± 0.4	2.4 ± 0.5	2.0 ± 1.2
CA mRNA/PGK1(×10^–2^)	11.1 ± 1	11.3 ± 0.8	9.8 ± 2	10.3 ± 2
DG mRNA/PGK1(×10^–2^)	11.5 ± 1.0	10.2 ± 0.8	7.8 ± 1.3*	10.1 ± 2.4*#
TrkB				
PVNmRNA/PGK1 (×10^–1^)	3.1 ± 0.08	3.4 ± 0.3	3.8 ± 0.4*	3.9 ± 0.06*
RVLM mRNA/PGK1 (×10^–1^)	1.6 ± 0.2	1.6 ± 0.2	1.6 ± 0.1	1.6 ± 0.2
CA mRNA/PGK1(×10^–1^)	2.2 ± 0.3	2.3 ± 0.5	2.9 ± 0.3*	2.9 ± 0.8*
DG mRNA/PGK1(×10^–1^)	3.3 ± 0.3	2.7 ± 0.8	3.6 ± 0.3*	3.7 ± 0.3*
FNDC5				
PVN mRNA/PGK1 (×10^–1^)	1.7 ± 0.3	1.6 ± 0.1	1.7 ± 0.3	1.7 ± 0.08
RVLM mRNA/PGK1 (×10^–1^)	3.0 ± 0.4	2.8 ± 0.3	2.8 ± 0.6	2.8 ± 0.4
CA mRNA/PGK1(×10^–1^)	3.4 ± 0.1	3.1 ± 0.6	3.2 ± 0.1	3.1 ± 0.5
DG mRNA/PGK1(×10^–1^)	2.6 ± 0.2	2.3 ± 0.5	2.5 ± 0.2	2.4 ± 0.2
AT_1_R				
PVN mRNA/PGK1 (×10^–2^)	3.2 ± 0.5	3.4 ± 0.8	3.5 ± 0.2	4.1 ± 0.5
RVLM mRNA/PGK1 (×10^–2^)	1.7 ± 0.1	1.5 ± 0.4	1.4 ± 0.3	1.4 ± 0.3
CA mRNA/PGK1(×10^–2^)	0.7 ± 0.4	0.7 ± 0.4	0.5 ± 0.2	0.6 ± 0.3
DG mRNA/PGK1(×10^–2^)	1.7 ± 0.2	1.4 ± 0.3	1.1 ± 0.1*	1.1 ± 0.4*
MR				
PVN mRNA/PGK1 (×10^–2^)	1.7 ± 0.3	1.4 ± 0.2	1.5 ± 0.2	1.5 ± 0.1
RVLM mRNA/PGK1 (×10^–2^)	2.2 ± 0.2	1.7 ± 0.4	1.4 ± 0.3*	1.3 ± 0.2*
CA mRNA/PGK1(×10^–2^)	11.9 ± 2.3	13.1 ± 3.3	12.9 ± 2.5	13.4 ± 3.1
DG mRNA/PGK1(×10^–2^)	12.7 ± 0.2	9.5 ± 0.3§	9.9 ± 0.1§	12.6 ± 0.2

Note

Values are means ± *SD* (*n* = 4/group). For BDNF in the PVN: SHR versus WKY, *F* = .12, *p* = .73 NS; ExT versus Sed, *F* = .65, *p* = .44 NS; ExT × strain interaction, *F* = 3.99, *p* = .07 NS. For BDNF in the RVLM: SHR versus WKY, *F* = 2.40, *p* = .15 NS; ExT versus Sed, *F* = .38, *p* = .55 NS; ExT × strain interaction, *F* = .53, *p* = .48 NS. For BDNF in the CA: SHR versus WKY, *F* = 2.18, *p* = .17 NS; ExT versus Sed, *F* = .22, *p* = .65 NS; ExT × strain interaction, *F* = .01, *p* = .92 NS. For BDNF in the DG: SHR versus WKY, *F* = 10.77, *p* = .0066; ExT versus Sed, *F* = .93, *p* = .35 NS; ExT × strain interaction, *F* = 6.75, *p* = .023. For TrkB in the PVN: SHR versus WKY, *F* = 22.27, *p* = .0005. ExT versus Sed, *F* = 3.08, *p* = .11 NS; ExT × strain interaction, *F* = .29, *p* = .60 NS. For TrkB in the RVLM: SHR versus WKY, *F* = .05, *p* = .83 NS; ExT versus Sed, *F* = .001, *p* = .97 NS; ExT × strain interaction, *F* = .07, *p* = .80 NS. For TrkB in the CA: SHR versus WKY, *F* = 5.90, *p* = .032; ExT versus Sed, *F* = .12, *p* = .74 NS; ExT × strain interaction, *F* = .06, *p* = .81 NS. For TrkB in the DG: SHR versus WKY, *F* = 7.02, *p* = .021; ExT versus Sed, *F* = .97, *p* = .34 NS; ExT × strain interaction, *F* = 2.44, *p* = .14 NS. For FNDC5 in the PVN: SHR versus WKY, *F* = .22, *p* = .65 NS; ExT versus Sed, *F* = .10, *p* = .76 NS; ExT × strain interaction, *F* = .16, *p* = .69 NS. For FNDC5 in the RVLM: SHR versus WKY, *F* = .31, *p* = .59 NS; ExT versus Sed, *F* = .36, *p* = .56 NS; ExT × strain interaction, *F* = .09, *p* = .77 NS. For FNDC5 in the CA: SHR versus WKY, *F* = .13, *p* = .73 NS; ExT versus Sed, *F* = 1.22, *p* = .29 NS; ExT × strain interaction, *F* = .21, *p* = .66 NS. For FNDC5 in the DG: SHR versus WKY, *F* = .004, *p* = .95 NS; ExT versus Sed, *F* = 1.53, *p* = .24 NS; ExT × strain interaction, *F* = .38, *p* = .55 NS. For AT_1_R in the PVN: SHR versus WKY, *F* = 3.40, *p* = .09 NS; ExT versus Sed, *F* = 1.60, *p* = .23 NS; ExT × strain interaction, *F* = .61, *p* = .45 NS. For AT_1_R in the RVLM: SHR versus WKY, *F* = 2.14, *p* = .17 NS; ExT versus Sed, *F* = .49, *p* = .50 NS; ExT × strain interaction, *F* = .25, *p* = .62 NS. For AT_1_R in the CA: SHR versus WKY, *F* = .97, *p* = .35 NS; ExT versus Sed, *F* = .06, *p* = .81 NS; ExT × strain interaction, *F* = .35, *p* = .57 NS. For AT_1_R in the DG: SHR versus WKY, *F* = 9.77, *p* = .0096; ExT versus Sed, *F* = .59, *p* = .46 NS; ExT × strain interaction, *F* = .87, *p* = .37 NS. For MR in the PVN: SHR versus WKY, *F* = .07, *p* = .79 NS; ExT versus Sed, *F* = 2.76, *p* = .12 NS; ExT × strain interaction, *F* = 2.12, *p* = .17 NS. For MR in the RVLM: SHR versus WKY, *F* = 15.68, *p* = .002; ExT versus Sed, *F* = 4.62, *p* = .05 NS; ExT × strain interaction, *F* = 2.56, *p* = .14 NS. For MR in the CA: SHR versus WKY, *F* = .20, *p* = .66 NS; ExT versus Sed, *F* = .38, *p* = .55 NS; ExT × strain interaction, *F* = .08, *p* = .78 NS. For MR in the DG: SHR versus WKY, *F* = .03, *p* = .87 NS; ExT versus Sed, *F* = .02, *p* = .90 NS; ExT × strain interaction, *F* = 6.85, *p* = .0225.

* *p* < .05 versus WKY.

^#^ *p* < .05 versus SHR‐Sed.

^§^ *p* < .05 versus WKY‐Sed or SHR‐ExT.

**Figure 3:**
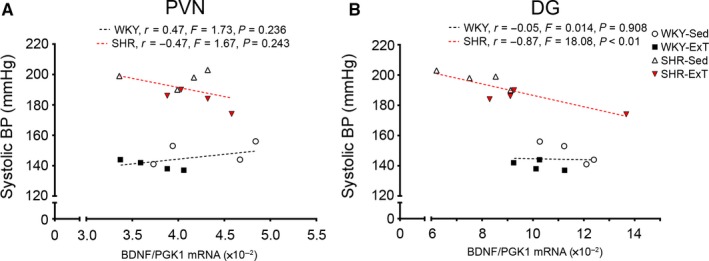
Correlations between blood pressure and BDNF gene expression in the brain. Correlations between systolic blood pressure versus relative BDNF mRNA expression in the (A) paraventricular nucleus (PVN), and (B) the dentate gyrus (DG) of the hippocampus. Gene expression is normalized to the housekeeping gene PGK1

**Figure 4:**
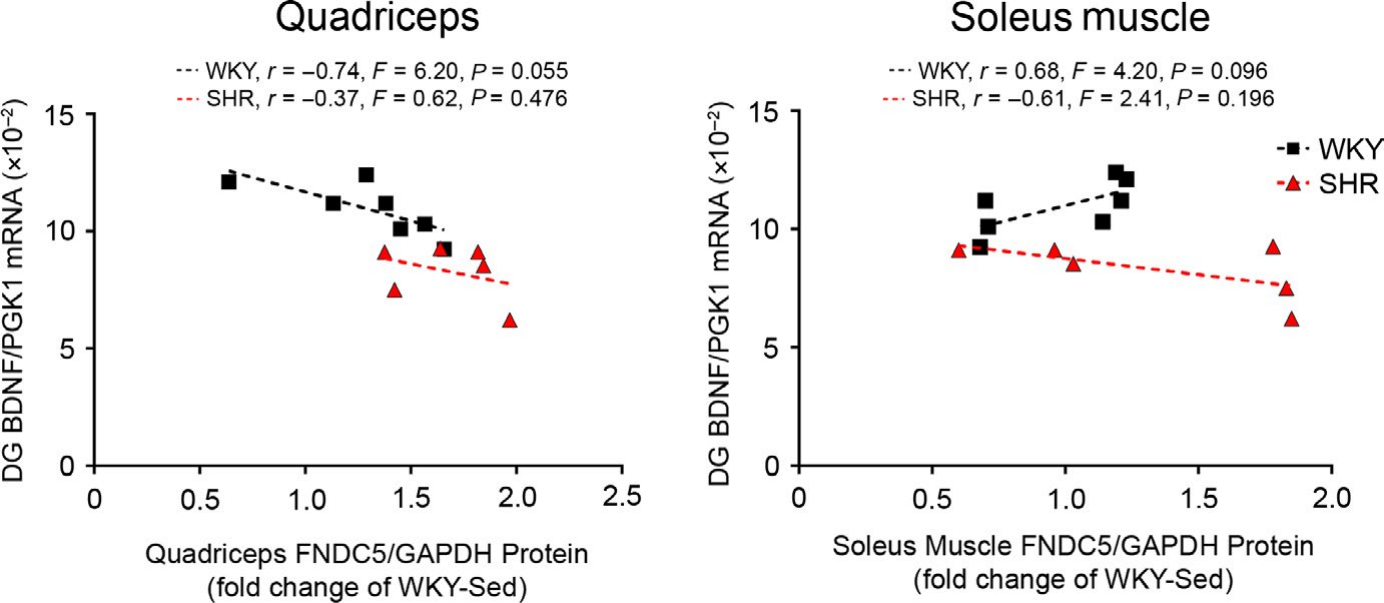
Correlations between skeletal muscle FNDC5 protein expression and hippocampal BDNF gene expression. Correlations between relative quadriceps or soleus muscle FNDC5 protein expression versus relative BDNF mRNA expression in the dentate gyrus (DG) of the hippocampus. Protein expression is normalized to housekeeping protein GAPDH and expressed as fold change of WKY‐Sed. Gene expression is normalized to the housekeeping gene PGK1

Given that hypertension is associated with neurocognitive deficits, we examined the expression of BDNF, TrkB, FNDC5, AT_1_R, and MR in the CA1‐3 and DG regions of the hippocampus. In the CA regions, TrkB expression was higher in SHR versus WKY, while BDNF, FNDC5, AT_1_R, and MR expressions were similar. Exercise did not affect BDNF, TrkB, FNDC5, AT_1_R, or MR expression. In the DG, BDNF was significantly lower in SHR‐Sed compared to WKY. BDNF expression in SHR‐ExT was significantly higher than SHR‐Sed, and 5 weeks of exercise training normalized BDNF deficits in SHR. Of note, relative BDNF expression in the DG negatively correlated with SBP in SHR (*r* = −.87, *p* < .01; Figure 3b), but not in WKY. Relative skeletal muscle FNDC5 protein tended to correlate with relative BDNF expression in the DG in WKY (quadriceps: *r* = −.74, *p* = .055; soleus muscle: *r* = .68, *p* = .096; Figure 4), but not SHR. TrkB expression was higher in SHR versus WKY, and unaffected by exercise. FNDC5 was similar between groups. AT_1_R was lower in SHR versus WKY, and not affected by exercise. MR expression was lower in SHR versus WKY, whereas exercise lowered MR in WKY but increased MR in SHR.

## 4 DISCUSSION

This study explored the effect of exercise on BDNF and FNDC5 in SHR versus WKY rats. Five weeks of moderate‐intensity treadmill exercise training—as expected—reduced SBP and DBP in both SHR and WKY. In the LV, BDNF protein expression was elevated in SHR and increased by exercise in both strains, while FNDC5 protein was also higher in SHR but unaffected by exercise. In skeletal muscle, FNDC5 protein was higher in SHR in both the quadriceps and soleus muscle, and exercise increased FNDC5 protein in the quadriceps. In the brain, BDNF expression in the PVN and RVLM were unaffected, but the expression of its receptor TrkB was significantly higher in SHR. Furthermore, hypertension was associated with significantly reduced BDNF mRNA in the DG of the hippocampus but not the CA regions, PVN, or RVLM. The reduced BDNF in SHR was normalized by exercise training. FNDC5 expression in these brain regions was similar between strains and unaffected by exercise training.

Our finding that 5 weeks of moderate intensity treadmill exercise reduced BP in both hypertensive and normotensive rats is consistent with other studies of similar type, intensity, and duration (Hsu et al., 2011; Lee et al., 2017, 2018; Petriz et al., 2015). A shorter (7 days) exercise training period did not elicit these changes (Monnier et al., 2017).

BDNF is highly expressed in the heart where it may serve cardioprotective functions, through, for example, modulating cardiomyocyte calcium cycling and promoting coronary angiogenesis (Kermani & Hempstead, 2019). Exercise promotes improved remodeling and function of the heart, in part by promoting the expression of BDNF (Lee et al., 2017, 2018). Indeed, BDNF protein (but not mRNA) expression was elevated in the LV of SHR, and following exercise, was increased in both hypertensive and normotensive strains. Amoureux et al. (2012) reported that aortic BDNF expression is elevated in SHR beginning at the development of hypertension (6 weeks of age) and continuing into adulthood (13 weeks of age). In contrast, Prigent‐Tessier et al. (2013) reported decreased mBDNF protein in the coronary endothelium and whole hearts of 10–11‐week‐old SHR versus WKY by immunohistochemistry and western blot respectively, and this was attenuated by 1 week of treadmill exercise training. We also observed elevated FNDC5 protein (but not mRNA) in the LV of SHR. While FNDC5 expression has not yet been examined in the hypertensive heart, Ling et al. (2018) reported decreased FNDC5 mRNA and protein in the aortas of 4 and 12‐week‐old SHR versus WKY, which was evident throughout the medial and adventitial layers, and FNDC5 over‐expression attenuated medial thickening in SHR. Yu et al. (2019) demonstrated that transverse aortic constriction‐induced hypertrophy upregulated FNDC5 protein in mice, which is similar to our finding. In our previous study, we reported decreases in FNDC5 mRNA and increases in FNDC5 protein in rats post‐MI (Lee et al., 2017). All together, these findings suggest that changes in FNDC5/BDNF signaling may play a role in cardiovascular pathologies such as hypertension or MI. Exercise did not affect FNDC5 expression in either group. Previously we reported that 5 weeks of exercise training also did not affect FNDC5 expression in the LV of rats with heart failure post‐MI (Lee et al., 2017). Collectively, our findings suggest that exercise‐mediated benefits occur independent of FNDC5 expression in the LV, regardless of cardiovascular pathology, and support the contribution of other sources of FNDC5, such as skeletal muscle.

The effect of hypertension on skeletal muscle BDNF expression has been largely unexplored. In this study, BDNF mRNA was elevated in the quadriceps of SHR with or without exercise training, whereas BDNF protein expression was moderately reduced in the quadriceps by exercise. Jimenez‐Maldonado et al. (2016) reported that 8 weeks of high‐intensity treadmill exercise, but not moderate‐intensity exercise, resulted in higher BDNF mRNA expression and trends toward lower BDNF protein in the soleus muscle, which may suggest that the intensity and duration of exercise affect the extent of BDNF expression. In addition, BDNF expression negatively correlates with myogenic differentiation from myocyte precursor cells, suggesting that lower BDNF expression may contribute to more myogenesis (Mousavi & Jasmin, 2006). FNDC5 mRNA was lower while FNDC5 protein was higher in the quadriceps of SHR, and FNDC5 protein was also higher in soleus muscle of SHR. Exercise increased FNDC5 protein expression in the quadriceps of both strains, which is consistent with previous studies; Wrann et al. (2013)*.* reported that increased FNDC5 expression may contribute to increased hippocampal BDNF via the secretion of irisin. Lecker et al. (2012) demonstrated that skeletal muscle FNDC5 gene expression positively correlates with improved cardiorespiratory capacity in heart failure patients.

In this study, we evaluated the effect of hypertension and exercise on two key functional regions in the brain: (a) cardiovascular regulatory centres involved in central control of BP, and (b) neurotrophin‐mediated cognition in the hippocampus. Hypertension and exercise had no effect on BDNF expression in the PVN or RVLM, but its receptor TrkB was upregulated in the PVN of SHR. Previous studies have demonstrated that BDNF mRNA in the PVN increases in response to salt‐induced osmotic stress (Aliaga, Arancibia, Givalois, & Tapia‐Arancibia, 2002) or immobilization‐induced psychological stress (Smith, Makino, Kim, & Kvetnansky, 1995). Acute and chronic BDNF elevation in the PVN increases BP in rats (Erdos et al., 2015; Schaich, Wellman, Koi, & Erdos, 2016). Complementing our finding that TrkB was elevated in SHR, Schaich et al. (2018) inhibited BDNF‐TrkB signaling by overexpressing a truncated‐form of TrkB (TrkB.T1) and observed a decrease in BP in rats, suggesting that TrkB contributes to BDNF‐TrkB signaling to raise BP. Assessing downstream angiotensinergic signaling in the PVN and RVLM, AT_1_R expression was unaffected in SHR or by exercise, while MR was decreased in the RVLM of SHR and unaffected by exercise. In contrast, Pietranera et al. (2012) reported higher MR mRNA expression in the PVN and hippocampus of SHR. Nakagaki et al. (2012) reported no difference in MR protein expression in the stroke‐prone SHR strain. The hippocampus is a critical site of neurogenesis and cognitive function, which may be disrupted in association with hypertension. Shih et al. (2016) reported decreased hippocampal BDNF expression, worse long‐term memory, and fewer BrdU‐incorporated DG cells suggesting less neuronal proliferation in mice subjected to a renal artery clip‐induced hypertension. This present study also observed decreased BDNF in the DG region. BDNF levels were restored by exercise training, which is consistent with previous work showing increased BDNF in the DG following exercise (Fang et al., 2013; Liu & Nusslock, 2018; Monnier et al., 2017). In contrast, TrkB was elevated in both the CA1‐3 and DG regions of SHR. FNDC5 expression in the PVN, RVLM, CA1‐3, or DG was unaffected in SHR or by exercise, suggesting that peripheral, not central, FNDC5 expression may contribute to FNDC5‐mediated regulation of BDNF.

In this study, we observed discrepancies between mRNA and protein expressions, suggesting the involvement of post‐transcriptional modulators such as microRNAs (miRNAs). miRNAs are small noncoding RNAs which repress gene translation by binding to mRNA transcripts and may affect protein production without affecting mRNA production (Zhou et al., 2018). Several miRNAs, such as microRNAs‐1 (Ma et al., 2015), −210 (Lin et al., 2010), −322 (Yang, Song, & Lv, 2016), are known to modulate BDNF expression in both cardiovascular and brain pathophysiology. Recently, microRNA‐135a‐5p was shown to affect FNDC5 expression (Metwally et al., 2019). Changes to the FNDC5 protein after translation may also account for discrepancies in mRNA and protein expression. Similarly, the conversion of proBDNF to mBDNF may account for differences in BDNF protein versus mRNA expression. ProBDNF levels were not examined in this study, but posits an area of future experimental examination.

Inflammation is associated with the onset of hypertension and its subsequent comorbidities such as neurological defects. Trends toward lower levels of pro‐inflammatory cytokine IL‐1α and higher levels of anti‐inflammatory cytokines IL‐4 and IL‐13 were found following exercise, although these did not reach statistical significance. IL‐4 and IL‐13 are known to be expressed and secreted by skeletal muscle in response to exercise training (Peake, Della Gatta, Suzuki, & Nieman, 2015; Suzuki, 2018) and contribute to the phenotypic switching of macrophages (including microglia in the brain) from a pro‐inflammatory M1 state to a reparative M2 state (Littlefield & Kohman, 2017). Exercise may also enhance clearance of neurotoxic compounds. The tryptophan‐kynurenine pathway produces neurotoxic metabolites which leads to N‐methyl‐D‐aspartate (NMDA) receptor activation, free radical production, and BDNF reduction (Vécsei, Szalárdy, Fülöp, & Toldi, 2013). In addition to inducing FNDC5, exercise‐induced skeletal muscle PGC‐1α increases aminotransferases which catalyzes the conversion of kynurenine to kynurenic acid (Agudelo et al., 2014), a form that cannot cross the blood‐brain barrier. This consequently protects the brain from neuro‐inflammation (Campbell, Charych, Lee, & Moller, 2014; Fukui, Schwarcz, Rapoport, Takada, & Smith, 1991) and prevents decreases in brain BDNF (Calabrese et al., 2014).

We identified several limitations to this study and potential future directions for further study. Firstly, changes in BDNF in SHR versus WKY may be due to strain differences, rather than hypertension per se, which may be delineated by, for example, the use of inhibitors of BDNF‐TrkB signaling. In addition, our assays do not discern between different cell types within the tissues of interest, and thus cannot account for heterogeneity in expression. For example, different cardiac cell types may be differentially affected by hypertension or exercise (Tao, Bei, Zhang, Xiao, & Li, 2015; Tirziu, Giordano, & Simons, 2010). Similarly, skeletal muscles differ in their proportion of oxidative and glycolytic muscle fibre types, and the brain consists of neurons and glia, all of which have been shown to have differing responses to exercise (Hyatt et al., 2015; Stevenson, Lensmire, & Swain, 2018 *)*. Further studies are required to determine cell type‐specific responses to hypertension or exercise. Lastly, this study reported moderate differences in circulating cytokines and plasma BDNF. The cellular sources of these plasma factors remain to be determined. Previous studies have suggested the brain as a major contributor of plasma BDNF after exercise training (Rasmussen et al., 2009; Seifert et al., 2010).

In summary, 5 weeks of treadmill exercise training reduced BP in both SHR and WKY. BDNF mRNA was increased in the quadriceps and BDNF protein was increased in the LV of SHR, while BDNF mRNA in the DG was lower in SHR, suggesting that hypertension increased BDNF expression in peripheral tissues while decreasing hippocampal BDNF. Exercise normalized BDNF mRNA in the DG of SHR, while BDNF protein was increased in the LV and decreased in the quadriceps of both strains. FNDC5 mRNA was lower in the LV while FNDC5 protein was higher in the LV, quadriceps, and soleus muscle of SHR. Exercise increased FNDC5 protein (but not mRNA) only in the quadriceps of both strains. FNDC5 in the heart and skeletal muscle appears involved in the response to hypertension, while only skeletal muscle is involved in the exercise response. No differences were observed in brain FNDC5, indicating that central FNDC5 is not involved in the response to hypertension or exercise. Thus, targeting the skeletal muscle FNDC5/BDNF pathway through endurance exercise may benefit the heart, skeletal muscle, and brain and lead to novel therapeutic approaches against hypertension and its associated comorbidities.

## CONFLICT OF INTEREST

The authors have no competing interests to declare.

## AUTHOR CONTRIBUTIONS

F.H.H.L. conceived and designed the study. T.W., M.T.M., H.W.L., M.A., and H.W.W. acquired the data. T.W. and M.T.M. drafted the manuscript. T.W., M.T.M., H.W.L., M.A., H.W.W., and F.H.H.L. analyzed and interpreted the data and critically revised the draft for intellectual content. All authors approved the final version of the manuscript and agree to be accountable for all aspects of the work in ensuring that questions related to the accuracy or integrity of any part of the work are appropriately investigated and resolved. All persons designated as authors qualify for authorship, and all those who qualify for authorship are listed.

## References

[phy214323-bib-0001] Agarwal, D. , Welsch, M. A. , Keller, J. N. , & Francis, J. (2011). Chronic exercise modulates RAS components and improves balance between pro‐ and anti‐inflammatory cytokines in the brain of SHR. Basic Research in Cardiology, 106, 1069–1085. 10.1007/s00395-011-0231-7 22124756 PMC3261080

[phy214323-bib-0002] Agudelo, L. Z. , Femenia, T. , Orhan, F. , Porsmyr‐Palmertz, M. , Goiny, M. , Martinez‐Redondo, V. , … Ruas, J. L. (2014). Skeletal muscle PGC‐1alpha1 modulates kynurenine metabolism and mediates resilience to stress‐induced depression. Cell, 159, 33–45. 10.1016/j.cell.2014.07.051 25259918

[phy214323-bib-0003] Aliaga, E. , Arancibia, S. , Givalois, L. , & Tapia‐Arancibia, L. (2002). Osmotic stress increases brain‐derived neurotrophic factor messenger RNA expression in the hypothalamic supraoptic nucleus with differential regulation of its transcrionts. Relation to arginine‐vasopressin content. Neuroscience, 112, 841–850. 10.1016/S0306-4522(02)00128-8 12088743

[phy214323-bib-0004] Amoureux, S. , Lorgis, L. , Sicard, P. , Girard, C. , Rochette, L. , & Vergely, C. (2012). Vascular BDNF expression and oxidative stress during aging and the development of chronic hypertension. Fundamental & Clinical Pharmacology, 26, 227–234. 10.1111/j.1472-8206.2010.00912.x 21210848

[phy214323-bib-0005] Becker, B. K. , Wang, H. J. , Tian, C. , & Zucker, I. H. (2015). BDNF contributes to angiotensin II‐mediated reductions in peak voltage‐gated K+ current in cultured CATH.a cells. Physiological Reports, 3, 1–8. 10.14814/phy2.12598 PMC4673628 26537343

[phy214323-bib-0006] Bostrom, P. , Wu, J. , Jedrychowski, M. P. , Korde, A. , Ye, L. , Lo, J. C. , … Spiegelman, B. M. (2012). A PGC1‐alpha‐dependent myokine that drives brown‐fat‐like development of white fat and thermogenesis. Nature, 481, 463–468. 10.1038/nature10777 22237023 PMC3522098

[phy214323-bib-0007] Calabrese, F. , Rossetti, A. C. , Racagni, G. , Gass, P. , Riva, M. A. , & Molteni, R. (2014). Brain‐derived neurotrophic factor: A bridge between inflammation and neuroplasticity. Frontiers in Cellular Neuroscience, 8, 1–7. 10.3389/fncel.2014.00430 25565964 PMC4273623

[phy214323-bib-0008] Campbell, B. M. , Charych, E. , Lee, A. W. , & Moller, T. (2014). Kynurenines in CNS disease: Regulation by inflammatory cytokines. Frontiers in Neuroscience, 8, 1–22. 10.3389/fnins.2014.00012 24567701 PMC3915289

[phy214323-bib-0009] Chan, S. H. , Wu, C. W. , Chang, A. Y. , Hsu, K. S. , & Chan, J. Y. (2010). Transcriptional upregulation of brain‐derived neurotrophic factor in rostral ventrolateral medulla by angiotensin II: Significance in superoxide homeostasis and neural regulation of arterial pressure. Circulation Research, 107, 1127–1139. 10.1161/CIRCRESAHA.110.225573 20814019

[phy214323-bib-0010] Chen, R. R. , Fan, X. H. , Chen, G. , Zeng, G. W. , Xue, Y. G. , Liu, X. T. , & Wang, C. Y. (2019). Irisin attenuates angiotensin II‐induced cardiac fibrosis via Nrf2 mediated inhibition of ROS/ TGFbeta1/Smad2/3 signaling axis. Chemico‐Biological Interactions, 302, 11–21. 10.1016/j.cbi.2019.01.031 30703374

[phy214323-bib-0011] Delezie, J. , & Handschin, C. (2018). Endocrine crosstalk between skeletal muscle and the brain. Frontiers in Neurology, 9, 1–14. 10.3389/fneur.2018.00698 30197620 PMC6117390

[phy214323-bib-0012] Erdos, B. , Backes, I. , McCowan, M. L. , Hayward, L. F. , & Scheuer, D. A. (2015). Brain‐derived neurotrophic factor modulates angiotensin signaling in the hypothalamus to increase blood pressure in rats. American Journal of Physiology. Heart and Circulatory Physiology, 308, H612–H622. 10.1152/ajpheart.00776.2014 25576628 PMC4360054

[phy214323-bib-0013] Fang, Z. H. , Lee, C. H. , Seo, M. K. , Cho, H. , Lee, J. G. , Lee, B. J. , … Kim, Y. H. (2013). Effect of treadmill exercise on the BDNF‐mediated pathway in the hippocampus of stressed rats. Neuroscience Research, 76, 187–194. 10.1016/j.neures.2013.04.005 23665137

[phy214323-bib-0014] Feng, N. , Huke, S. , Zhu, G. , Tocchetti, C. G. , Shi, S. , Aiba, T. , … Paolocci, N. (2015). Constitutive BDNF/TrkB signaling is required for normal cardiac contraction and relaxation. Proceedings of the National Academy of Sciences, 112, 1880–1885. 10.1073/pnas.1417949112 PMC4330748 25583515

[phy214323-bib-0015] Fiuza‐Luces, C. , Santos‐Lozano, A. , Joyner, M. , Carrera‐Bastos, P. , Picazo, O. , Zugaza, J. L. , … Lucia, A. (2018). Exercise benefits in cardiovascular disease: Beyond attenuation of traditional risk factors. Nature Reviews Cardiology, 15, 731–743. 10.1038/s41569-018-0065-1 30115967

[phy214323-bib-0016] Fu, J. , Han, Y. , Wang, J. , Liu, Y. , Zheng, S. , Zhou, L. , … Zeng, C. (2016). Irisin Lowers blood pressure by improvement of endothelial dysfunction via AMPK‐Akt‐eNOS‐NO pathway in the spontaneously hypertensive rat. Journal of the American Heart Association, 5, 1–12. 10.1161/JAHA.116.003433 PMC5210324 27912206

[phy214323-bib-0017] Fukui, S. , Schwarcz, R. , Rapoport, S. I. , Takada, Y. , & Smith, Q. R. (1991). Blood‐brain barrier transport of kynurenines: Implications for brain synthesis and metabolism. Journal of Neurochemistry, 56, 2007–2017. 10.1111/j.1471-4159.1991.tb03460.x 1827495

[phy214323-bib-0018] Garcia, C. , Chen, M. J. , Garza, A. A. , Cotman, C. W. , & Russo‐Neustadt, A. (2003). The influence of specific noradrenergic and serotonergic lesions on the expression of hippocampal brain‐derived neurotrophic factor transcripts following voluntary physical activity. Neuroscience, 119, 721–732. 10.1016/S0306-4522(03)00192-1 12809693

[phy214323-bib-0019] Greenberg, M. E. , Xu, B. , Lu, B. , & Hempstead, B. L. (2009). New insights in the biology of BDNF synthesis and release: Implications in CNS function. Journal of Neuroscience, 29, 12764–12767. 10.1523/JNEUROSCI.3566-09.2009 19828787 PMC3091387

[phy214323-bib-0020] Hsu, Y. C. , Chen, H. I. , Kuo, Y. M. , Yu, L. , Huang, T. Y. , Chen, S. J. , … Jen, C. J. (2011). Chronic treadmill running in normotensive rats resets the resting blood pressure to lower levels by upregulating the hypothalamic GABAergic system. Journal of Hypertension, 29, 2339–2348. 10.1097/HJH.0b013e32834c628f 22002337

[phy214323-bib-0021] Hyatt, H. W. , Toedebusch, R. G. , Ruegsegger, G. , Mobley, C. B. , Fox, C. D. , McGinnis, G. R. , … Kavazis, A. N. (2015). Comparative adaptations in oxidative and glycolytic muscle fibers in a low voluntary wheel running rat model performing three levels of physical activity. Physiological Reports, 3, 1–11. 10.14814/phy2.12619 PMC4673647 26603455

[phy214323-bib-0022] Jimenez‐Maldonado, A. , Cerna‐Cortes, J. , Castro‐Rodriguez, E. M. , Montero, S. A. , Muniz, J. , Rodriguez‐Hernandez, A. , … De Alvarez‐Buylla, E. R. (2016). Effects of moderate‐ and high‐intensity chronic exercise on brain‐derived neurotrophic factor expression in fast and slow muscles. Muscle and Nerve, 53, 446–451. 10.1002/mus.24757 26148339

[phy214323-bib-0023] Kermani, P. , & Hempstead, B. (2019). BDNF actions in the cardiovascular system: roles in development, adulthood and response to injury. Frontiers in Physiology, 10, 1–8. 10.3389/fphys.2019.00455 31105581 PMC6498408

[phy214323-bib-0024] Lavie, C. J. , Arena, R. , Swift, D. L. , Johannsen, N. M. , Sui, X. , Lee, D. C. , … Blair, S. N. (2015). Exercise and the cardiovascular system: Clinical science and cardiovascular outcomes. Circulation Research, 117, 207–219. 10.1161/CIRCRESAHA.117.305205 26139859 PMC4493772

[phy214323-bib-0025] Lear, S. A. , Hu, W. , Rangarajan, S. , Gasevic, D. , Leong, D. , Iqbal, R. , … Yusuf, S. (2017). The effect of physical activity on mortality and cardiovascular disease in 130 000 people from 17 high‐income, middle‐income, and low‐income countries: The PURE study. The Lancet, 390, 2643–2654. 10.1016/S0140-6736(17)31634-3 28943267

[phy214323-bib-0026] Lecker, S. H. , Zavin, A. , Cao, P. , Arena, R. , Allsup, K. , Daniels, K. M. , … Forman, D. E. (2012). Expression of the irisin precursor FNDC5 in skeletal muscle correlates with aerobic exercise performance in patients with heart failure. Circulation: Heart Failure, 5, 812–818. 10.1161/CIRCHEARTFAILURE.112.969543 23001918 PMC7284011

[phy214323-bib-0027] Lee, H. W. , Ahmad, M. , Wang, H. W. , & Leenen, F. H. (2017). Effects of exercise training on brain‐derived neurotrophic factor in skeletal muscle and heart of rats post myocardial infarction. Experimental Physiology, 102, 314–328. 10.1113/EP086049 28070911

[phy214323-bib-0028] Lee, H. W. , Ahmad, M. , Weldrick, J. J. , Wang, H.‐W. , Burgon, P. G. , & Leenen, F. H. H. (2018). Effects of exercise training and TrkB blockade on cardiac function and BDNF‐TrkB signaling postmyocardial infarction in rats. American Journal of Physiology. Heart and Circulatory Physiology, 315, H1821–H1834. 10.1152/ajpheart.00245.2018 30311496

[phy214323-bib-0029] Lee, P. , Linderman, J. D. , Smith, S. , Brychta, R. J. , Wang, J. , Idelson, C. , … Celi, F. S. (2014). Irisin and FGF21 are cold‐induced endocrine activators of brown fat function in humans. Cell Metabolism, 19, 302–309. 10.1016/j.cmet.2013.12.017 24506871 PMC7647184

[phy214323-bib-0030] Liao, Q. , Qu, S. , Tang, L. X. , Li, L. P. , He, D. F. , Zeng, C. Y. , & Wang, W. E. (2019). Irisin exerts a therapeutic effect against myocardial infarction via promoting angiogenesis. Acta Pharmacologica Sinica, 40, 1314–1321. 10.1038/s41401-019-0230-z 31061533 PMC6786355

[phy214323-bib-0031] Lin, R. C. , Weeks, K. L. , Gao, X. M. , Williams, R. B. , Bernardo, B. C. , Kiriazis, H. , … McMullen, J. R. (2010). PI3K(p110 alpha) protects against myocardial infarction‐induced heart failure: Identification of PI3K‐regulated miRNA and mRNA. Arteriosclerosis, Thrombosis, and Vascular Biology, 30, 724–732. 10.1161/ATVBAHA.109.201988 20237330

[phy214323-bib-0032] Ling, L. , Chen, D. , Tong, Y. , Zang, Y. H. , Ren, X. S. , Zhou, H. , … Zhu, G. Q. (2018). Fibronectin type III domain containing 5 attenuates NLRP3 inflammasome activation and phenotypic transformation of adventitial fibroblasts in spontaneously hypertensive rats. Journal of Hypertension, 36, 1104–1114. 10.1097/HJH.0000000000001654 29303830

[phy214323-bib-0033] Littlefield, A. , & Kohman, R. A. (2017). Differential response to intrahippocampal interleukin‐4/interleukin‐13 in aged and exercise mice. Neuroscience, 343, 106–114. 10.1016/j.neuroscience.2016.11.027 27916728 PMC5800496

[phy214323-bib-0034] Liu, P. Z. , & Nusslock, R. (2018). Exercise‐mediated neurogenesis in the hippocampus via BDNF. Frontiers in Neuroscience, 12, 1–6. 10.3389/fnins.2018.00052 29467613 PMC5808288

[phy214323-bib-0035] Ma, J. C. , Duan, M. J. , Sun, L. L. , Yan, M. L. , Liu, T. , Wang, Q. , … Ai, J. (2015). Cardiac over‐expression of microRNA‐1 induces impairment of cognition in mice. Neuroscience, 299, 66–78. 10.1016/j.neuroscience.2015.04.061 25943483

[phy214323-bib-0036] Matthews, V. B. , Åström, M.‐B. , Chan, M. H. S. , Bruce, R. , Krabbe, K. S. , Prelovsek, O. , … Febbraio, M. A. (2009). Brain‐derived neurotrophic factor is produced by skeletal muscle cells in response to contraction and enhances fat oxidation via activation of AMP‐activated protein kinase. Diabetologia, 52, 1409–1418. 10.1007/s00125-009-1364-1 19387610

[phy214323-bib-0037] Metwally, M. , Bayoumi, A. , Romero‐Gomez, M. , Thabet, K. , John, M. , Adams, L. A. , … Eslam, M. (2019). A polymorphism in the Irisin‐encoding gene (FNDC5) associates with hepatic steatosis by differential miRNA binding to the 3'UTR. Journal of Hepatology, 70, 494–500. 10.1016/j.jhep.2018.10.021 30389552

[phy214323-bib-0038] Monnier, A. , Garnier, P. , Quirie, A. , Pernet, N. , Demougeot, C. , Marie, C. , & Prigent‐Tessier, A. (2017). Effect of short‐term exercise training on brain‐derived neurotrophic factor signaling in spontaneously hypertensive rats. Journal of Hypertension, 35, 279–290. 10.1097/HJH.0000000000001164 28005701

[phy214323-bib-0039] Mousavi, K. , & Jasmin, B. J. (2006). BDNF is expressed in skeletal muscle satellite cells and inhibits myogenic differentiation. Journal of Neuroscience, 26, 5739–5749. 10.1523/JNEUROSCI.5398-05.2006 16723531 PMC6675269

[phy214323-bib-0040] Nakagaki, T. , Hirooka, Y. , Matsukawa, R. , Nishihara, M. , Nakano, M. , Ito, K. , … Sunagawa, K. (2012). Activation of mineralocorticoid receptors in the rostral ventrolateral medulla is involved in hypertensive mechanisms in stroke‐prone spontaneously hypertensive rats. Hypertension Research, 35, 470–476. 10.1038/hr.2011.220 22237482

[phy214323-bib-0041] Park, H. , & Poo, M. M. (2013). Neurotrophin regulation of neural circuit development and function. Nature Reviews Neuroscience, 14, 7–23. 10.1038/nrn3379 23254191

[phy214323-bib-0042] Paxinos, G. , & Watson, C. (1998). The rat brain in stereotaxic coordinates, 4th ed. Cambridge, MA: Academic Press.

[phy214323-bib-0043] Peake, J. M. , Della Gatta, P. , Suzuki, K. , & Nieman, D. C. (2015). Cytokine expression and secretion by skeletal muscle cells: Regulatory mechanisms and exercise effects. Exercise Immunology Review, 21, 8–25. 25826432

[phy214323-bib-0044] Petriz, B. A. , Almeida, J. A. , Gomes, C. P. , Ernesto, C. , Pereira, R. W. , & Franco, O. L. (2015). Exercise performed around MLSS decreases systolic blood pressure and increases aerobic fitness in hypertensive rats. BMC Physiology, 15, 1–6. 10.1186/s12899-015-0015-7 25888441 PMC4367833

[phy214323-bib-0045] Pietranera, L. , Brocca, M. E. , Cymeryng, C. , Gomez‐Sanchez, E. , Gomez‐Sanchez, C. E. , Roig, P. , … De Nicola, A. F. (2012). Increased expression of the mineralocorticoid receptor in the brain of spontaneously hypertensive rats. Journal of Neuroendocrinology, 24, 1249–1258. 10.1111/j.1365-2826.2012.02332.x 22564091

[phy214323-bib-0046] Pius‐Sadowska, E. , & Machalinski, B. (2017). BDNF – A key player in cardiovascular system. Journal of Molecular and Cellular Cardiology, 110, 54–60. 10.1016/j.yjmcc.2017.07.007 28736262

[phy214323-bib-0047] Prigent‐Tessier, A. , Quirie, A. , Maguin‐Gate, K. , Szostak, J. , Mossiat, C. , Nappey, M. , … Demougeot, C. (2013). Physical training and hypertension have opposite effects on endothelial brain‐derived neurotrophic factor expression. Cardiovascular Research, 100, 374–382. 10.1093/cvr/cvt219 24092446

[phy214323-bib-0048] Rasmussen, P. , Brassard, P. , Adser, H. , Pedersen, M. V. , Leick, L. , Hart, E. , … Pilegaard, H. (2009). Evidence for a release of brain‐derived neurotrophic factor from the brain during exercise. Experimental Physiology, 94, 1062–1069. 10.1113/expphysiol.2009.048512 19666694

[phy214323-bib-0049] Schaich, C. L. , Wellman, T. L. , Einwag, Z. , Dutko, R. A. , & Erdos, B. (2018). Inhibition of BDNF signaling in the paraventricular nucleus of the hypothalamus lowers acute stress‐induced pressor responses. Journal of Neurophysiology, 120, 633–643. 10.1152/jn.00459.2017 29694277 PMC6139453

[phy214323-bib-0050] Schaich, C. L. , Wellman, T. L. , Koi, B. , & Erdos, B. (2016). BDNF acting in the hypothalamus induces acute pressor responses under permissive control of angiotensin II. Autonomic Neuroscience, 197, 1–8. 10.1016/j.autneu.2016.02.011 26948539 PMC9387676

[phy214323-bib-0051] Seifert, T. , Brassard, P. , Wissenberg, M. , Rasmussen, P. , Nordby, P. , Stallknecht, B. , … Secher, N. H. (2010). Endurance training enhances BDNF release from the human brain. American Journal of Physiology: Regulatory, Integrative and Comparative Physiology, 298, R372–R377. 10.1152/ajpregu.00525.2009 19923361

[phy214323-bib-0052] Shih, Y. H. , Tsai, S. F. , Huang, S. H. , Chiang, Y. T. , Hughes, M. W. , Wu, S. Y. , … Kuo, Y. M. (2016). Hypertension impairs hippocampus‐related adult neurogenesis, CA1 neuron dendritic arborization and long‐term memory. Neuroscience, 322, 346–357. 10.1016/j.neuroscience.2016.02.045 26921651

[phy214323-bib-0053] Smith, M. A. , Makino, S. , Kim, S.‐Y. , & Kvetnansky, R. (1995). Stress increases brain‐derived neurotropic factor messenger ribonucleic acid in the hypothalamus and pituitary. Endocrinology, 136, 3743–3750. 10.1210/endo.136.9.7649080 7649080

[phy214323-bib-0054] Stevenson, M. E. , Lensmire, N. A. , & Swain, R. A. (2018). Astrocytes and radial glia‐like cells, but not neurons, display a nonapoptotic increase in caspase‐3 expression following exercise. Brain and Behavior, 8, 1–12. 10.1002/brb3.1110 PMC6192401 30240148

[phy214323-bib-0055] Suzuki, K. (2018). Cytokine response to exercise and its modulation. Antioxidants, 7, 1–7. 10.3390/antiox7010017

[phy214323-bib-0056] Tao, L. , Bei, Y. , Zhang, H. , Xiao, J. , & Li, X. (2015). Exercise for the heart: Signaling pathways. Oncotarget, 6, 20773–20784. 10.18632/oncotarget.4770 26318584 PMC4673228

[phy214323-bib-0057] Tirziu, D. , Giordano, F. J. , & Simons, M. (2010). Cell communications in the heart. Circulation, 122, 928–937. 10.1161/CIRCULATIONAHA.108.847731 20805439 PMC2941440

[phy214323-bib-0058] Vécsei, L. , Szalárdy, L. , Fülöp, F. , & Toldi, J. (2013). Kynurenines in the CNS: Recent advances and new questions. Nature Reviews Drug Discovery, 12, 64–82. 10.1038/nrd3793 23237916

[phy214323-bib-0059] Walsh, J. J. , & Tschakovsky, M. E. (2018). Exercise and circulating BDNF: Mechanisms of release and implications for the design of exercise interventions. Applied Physiology, Nutrition and Metabolism, 43, 1095–1104. 10.1139/apnm-2018-0192 29775542

[phy214323-bib-0060] Wang, H. W. , Amin, M. S. , El‐Shahat, E. , Huang, B. S. , Tuana, B. S. , & Leenen, F. H. (2010). Effects of central sodium on epithelial sodium channels in rat brain. American Journal of Physiology: Regulatory, Integrative and Comparative Physiology, 299, R222–R233. 10.1152/ajpregu.00834.2009 20427723

[phy214323-bib-0061] Wang, H. W. , Huang, B. S. , Chen, A. , Ahmad, M. , White, R. A. , & Leenen, F. H. (2016). Role of brain aldosterone and mineralocorticoid receptors in aldosterone‐salt hypertension in rats. Neuroscience, 314, 90–105. 10.1016/j.neuroscience.2015.11.055 26656220

[phy214323-bib-0062] Wrann, C. D. , White, J. P. , Salogiannnis, J. , Laznik‐Bogoslavski, D. , Wu, J. , Ma, D. , … Spiegelman, B. M. (2013). Exercise induces hippocampal BDNF through a PGC‐1alpha/FNDC5 pathway. Cell Metabolism, 18, 649–659. 10.1016/j.cmet.2013.09.008 24120943 PMC3980968

[phy214323-bib-0063] Yang, L. , Song, S. , & Lv, H. (2016). MicroRNA‐322 protects hypoxia‐induced apoptosis in cardiomyocytes via BDNF gene. American Journal of Translational Research, 8, 2812–2819. 27398164 PMC4931175

[phy214323-bib-0064] Yu, Q. , Kou, W. , Xu, X. , Zhou, S. , Luan, P. , Xu, X. , … Peng, W. (2019). FNDC5/Irisin inhibits pathological cardiac hypertrophy. Clinical Science, 133, 611–627. 10.1042/CS20190016 30782608

[phy214323-bib-0065] Yu, T. , Chang, Y. , Gao, X. L. , Li, H. , & Zhao, P. (2017). Dynamic expression and the role of BDNF in exercise‐induced skeletal muscle regeneration. International Journal of Sports Medicine, 38, 959–966. 10.1055/s-0043-118343 28965341

[phy214323-bib-0066] Zheng, H. , Sharma, N. M. , Liu, X. , & Patel, K. P. (2012). Exercise training normalizes enhanced sympathetic activation from the paraventricular nucleus in chronic heart failure: Role of angiotensin II. American Journal of Physiology: Regulatory, Integrative and Comparative Physiology, 303, R387–R394. 10.1152/ajpregu.00046.2012 PMC3423995 22718804

[phy214323-bib-0067] Zhou, S. S. , Jin, J. P. , Wang, J. Q. , Zhang, Z. G. , Freedman, J. H. , Zheng, Y. , & Cai, L. (2018). miRNAS in cardiovascular diseases: Potential biomarkers, therapeutic targets and challenges. Acta Pharmacologica Sinica, 39, 1073–1084. 10.1038/aps.2018.30 29877320 PMC6289363

[phy214323-bib-0068] Zuccato, C. , & Cattaneo, E. (2009). Brain‐derived neurotrophic factor in neurodegenerative diseases. Nature Reviews Neurology, 5, 311–322. 10.1038/nrneurol.2009.54 19498435

